# Use of diffusion spectrum imaging in preliminary longitudinal evaluation of amyotrophic lateral sclerosis: development of an imaging biomarker

**DOI:** 10.3389/fnhum.2014.00270

**Published:** 2014-04-29

**Authors:** Kumar Abhinav, Fang-Cheng Yeh, Ahmed El-Dokla, Lisa M. Ferrando, Yue-Fang Chang, David Lacomis, Robert M. Friedlander, Juan C. Fernandez-Miranda

**Affiliations:** ^1^Department of Neurological Surgery, University of Pittsburgh School of Medicine, University of Pittsburgh Medical Center, PittsburghPA, USA; ^2^Department of Biomedical Engineering, Carnegie Mellon University, PittsburghPA, USA; ^3^Department of Neurology, University of Pittsburgh Medical CenterPittsburgh, PA, USA; ^4^Walter Dandy Endowed Professor of Neurosurgery and Neurobiology, University of Pittsburgh School of Medicine, University of Pittsburgh Medical CenterPittsburgh, PA, USA

**Keywords:** diffusion spectrum imaging, amyotrophic lateral sclerosis, white matter, longitudinal, motoric, extramotoric

## Abstract

Previous diffusion tensor imaging (DTI) studies have shown white matter pathology in amyotrophic lateral sclerosis (ALS), predominantly in the motor pathways. Further these studies have shown that DTI can be used longitudinally to track pathology over time, making white matter pathology a candidate as an outcome measure in future trials. DTI has demonstrated application in group studies, however its derived indices, for example fractional anisotropy, are susceptible to partial volume effects, making its role questionable in examining individual progression. We hypothesize that changes in the white matter are present in ALS beyond the motor tracts, and that the affected pathways and associated pattern of disease progression can be tracked longitudinally using automated diffusion connectometry analysis. Connectometry analysis is based on diffusion spectrum imaging and overcomes the limitations of a conventional tractography approach and DTI. The identified affected white matter tracts can then be assessed in a targeted fashion using High definition fiber tractography (a novel white matter MR imaging technique). Changes in quantitative and qualitative markers over time could then be correlated with clinical progression. We illustrate these principles toward developing an imaging biomarker for demonstrating individual progression, by presenting results for five ALS patients, including with longitudinal data in two. Preliminary analysis demonstrated a number of changes bilaterally and asymmetrically in motoric and extramotoric white matter pathways. Further the limbic system was also affected possibly explaining the cognitive symptoms in ALS. In the two longitudinal subjects, the white matter changes were less extensive at baseline, although there was evidence of disease progression in a frontal pattern with a relatively spared postcentral gyrus, consistent with the known pathology in ALS.

## INTRODUCTION

Amyotrophic lateral sclerosis (ALS) is a neurodegenerative disorder, marked by progressive failure of both upper (UMN) and lower motor neurons (LMN). LMN symptoms in ALS may obscure the UMN symptoms, contributing at least partly to the potential delay in diagnosis and the subsequent treatment. An imaging biomarker would therefore be useful for diagnostic and monitoring purposes, especially for evaluating efficacy of treatments in future trials. Previous diffusion tensor imaging (DTI) studies have shown white matter pathology in ALS, predominantly in the motor pathways. Further these studies have shown that DTI can be used longitudinally to track pathology over time, making white matter pathology a candidate as an outcome measure in future trials. DTI (Jacob et al., 2003; Graham et al., 2004; Sach et al., 2004; Nickerson et al., 2009; Iwata et al., 2011; van der Graaff et al., 2011; Menke et al., 2012) studies in ALS have therefore been carried out to investigate the involvement of the white matter tracts and their potential correlation with clinical markers of progression like the revised ALS functional rating scale (ALSFRS-R; Cedarbaum et al., 1999; Brooks et al., 2000). These studies have relied on DTI-based indices [like fractional anisotropy (FA) or apparent diffusion coefficient (ADC)] for quantification of the observed changes in the integrity of white matter tracts. Corticospinal tract (CST) or corpus callosum (CC) have been of particular interest. In general, these studies have demonstrated a reduction in the values of FA, when compared to controls or when carried out in a longitudinal fashion (Jacob et al., 2003; Sage et al., 2007; Nickerson et al., 2009; Keil et al., 2012) over time along the CST. These studies have indirectly demonstrated disease progression in ALS. FA is a commonly used DTI-derived quantitative measure of anisotropy (Basser and Pierpaoli, 1996; Oouchi et al., 2007) and has been applied in the evaluation of white matter integrity in multiple neurological disease states including multiple sclerosis (Guo et al., 2002) and stroke (Thomalla et al., 2004). It has been suggested that the changes in FA may reflect changes in organization of fibers (Beaulieu, 2002) with a reduction reflecting axonal fiber degeneration and myelin breakdown in the central and peripheral nervous system (Beaulieu et al., 1996; Ciccarelli et al., 2006).

It is noteworthy that extramotoric changes have been demonstrated in multiple ALS studies, including in CC (Filippini et al., 2010); cingulum and hippocampal formation (Raaphorst et al., 2011) and cerebellum (Keil et al., 2012). One pathological study in ALS (Smith, 1960), based on post-mortem examination of seven patients, showed evidence of degenerating fibers in the pre-central gyrus (and CST), postcentral gyrus and in the adjacent frontal and parietal gyri, CC and basal ganglia. These changes suggest a multisystem neurodegenerative process. DTI and voxel based analysis (VBA) have been used in a complementary fashion to elucidate these changes to varying extents (Sage et al., 2007; van der Graaff et al., 2011; Rose et al., 2012; Verstraete et al., 2013). Although these studies have been promising in demonstrating white matter changes in ALS, DTI is acknowledged to have major limitations. These limitations include inability to represent the crossing of multiple fibers, the absence of large segments of tracts and other minor artifacts (Alexander and Barker, 2005; Le Bihan et al., 2006). The indices derived from DTI, for example FA, have been shown to suffer from the partial volume effects, which lead to their underestimation in regions containing crossing fibers (Alexander et al., 2001; Oouchi et al., 2007). This therefore, limits the use of FA as a quantitative marker in ALS. Other limitations pertinent to all tractography-based studies, are the inherent variability-particularly between observers in the tracts due to issues related to the regions of interest (ROI) chosen-and the manual nature of the analysis. VBA addresses some of these limitations; however it does not provide directional information in terms of the specific tracts involved. Due to these limitations, new fiber mapping techniques were developed such as high-angular-resolution diffusion imaging (HARDI; Tuch et al., 2002) and diffusion spectrum imaging (DSI). DSI measures diffusion spectra and enables resolution of intravoxel heterogeneity of diffusion. This results in the resolution of a set of directions of multiple pathways or fiber orientations within a voxel (Wedeen et al., 2005) in contrast to the average direction in DTI.

Our group has developed and demonstrated the use of an advanced white matter imaging technique, high definition fiber tractography (HDFT). HDFT addresses the limitations associated with DTI, and is accurate in tracking white fiber pathways in the brain through complex fiber crossings to subcortical regions and targets with subvoxel resolution (Fernandez-Miranda et al., 2012). HDFT relies on DSI for acquisition (Wedeen et al., 2005); generalized q-sampling imaging for fiber orientation estimation (Yeh et al., 2010); multiple intravoxel sampling; and other innovations in tractography (Verstynen et al., 2011). HDFT approach has facilitated innovative studies on the structural connectivity of several fiber tracts such as the middle longitudinal fascicle (Wang et al., 2013); CST (Verstynen et al., 2011); CC (Jarbo et al., 2012); dorsal stream visual pathways (Greenberg et al., 2012); and the corticostriatal pathways (Verstynen et al., 2012). Application of HDFT has enabled us to replicate several known neuroanatomical features, including claustrum, thalamic nuclei segmentation and multiple fiber crossings at the centrum semiovale (Fernandez-Miranda et al., 2012). Ongoing trials at our institution are examining its application in evaluating white fiber pathology in other diseases, for example cavernomas and brain tumors with promising preliminary results (Fernandez-Miranda et al., 2012). Quantitative anisotropy (QA) is our novel quantitative imaging measure related to our white matter imaging technique. QA is a measure of anisotropy of water with estimated spin density. QA serves as a reliable stopping criterion for fiber tracking and is essential toward demonstrating endpoint connectivity of functional regions (Yeh et al., 2010; Yeh et al., 2013b). It overcomes some of the limitations of FA in assessing the fiber integrity in areas with multiple crossing fibers, and is less sensitive to partial volume effects, as demonstrated in our recent study (Yeh et al., 2013b). As a directionally specific quantitative measure, it is ideal for longitudinally assessing fiber integrity in motoric and extramotoric white matter pathways in ALS patients.

Our specific hypotheses are as follows: changes in the white matter are present in ALS patients beyond the motor tracts; compromise of structural integrity of the white matter tracts can be demonstrated qualitatively and be indirectly measured by our imaging quantitative markers; and finally progression in affected white matter tracts correlates with clinical progression. Accordingly, our specific aim is to identify and track the affected white matter pathways and their longitudinal progression in a reliable manner, using automated diffusion connectometry analysis. We will then aim to further longitudinally evaluate identified affected white matter tracts, using HDFT and a targeted tractography approach. The latter will enable us to examine potential correlation between associated quantitative parameters, such as changes in QA and/or volume and clinical deterioration in ALS patients over time, for example as measured by ALSFRS-R. The overall aim using this methodology is to establish a reproducible imaging biomarker, which can identify and track progression of white matter changes in ALS patients.

## METHODS

### CLINICAL INCLUSION CRITERIA AND EVALUATION

We present our preliminary results with respect to five patients recruited into the present study. Two patients with probable ALS; two patients with probable ALS (laboratory supported) and one with possible ALS (predominantly UMN signs) were included in the present study. ALS patients were identified by the El-Escorial criteria (Brooks et al., 2000). Two patients have been scanned longitudinally, while the other three have had a baseline scan only with planned longitudinal scans in the future. Of two patients with longitudinal scans, patients 1 and 2 underwent two scans approximately 1 year and 6 months apart respectively.

Our study plan involves recruiting a total of 20 patients with ALS, including the diagnostic categories, as defined by the revised El-Escorial criteria (Brooks et al., 2000) of clinically definite; probable; probable-laboratory supported and possible ALS. A smaller subset with primary lateral sclerosis (PLS; Gordon et al., 2006) will also be included. Over a period of 2 years, these patients will undergo three scans, 6 months apart longitudinally. Corresponding to each scan session, the patients will also undergo extensive clinical evaluation and scoring including that of ALSFRS-R (Brooks et al., 2000), frontotemporal dementia assessment, forced vital capacity (FVC) and Ashworth spasticity scale as part of an ALS multidisciplinary clinic. Clinical evaluation will be carried out by two neurologists, with specialist interest in neuromuscular disorders. 20 age-matched neurologically normal controls will also be scanned three times in a similar fashion, in order to contribute to the matched template file for comparison for diffusion connectometry analysis. The data acquired from scanning the controls will also allow a direct matched comparison for targeted tractography in terms of changes in QA and/or volume for specific affected fiber tracts of interest, as identified by the connectometry analysis. Approval has been obtained from the institutional review board (IRB#: REN12100240/PRO09010491).

### TWO-STEP APPROACH TOWARD EVALUATING WHITE MATTER DISEASE IN ALS

We propose and have currently adopted a two-step approach to evaluating white matter disease in a longitudinal fashion in ALS patients with the first *primary* step involving diffusion connectometry (Yeh et al., 2013a) analysis to identify the affected white matter tracts followed by a detailed tractography approach toward the evaluation of these identified abnormal fiber pathways. Diffusion connectometry (Yeh et al., 2013a) is a fully automatic method for examining fiber tracts. It uses the diffusion MRI data of normal subjects as a norm to statistically examine pathways with deviant or abnormal connectivity in affected individuals. We have previously demonstrated its utility in seven patients with chronic stroke and compared the results with lesions shown on T_2_-weighted images; ADC and FA maps as well as clinical manifestations. The results were noteworthy in demonstrating that the affected tracts revealed by connectometry corresponded well with the stroke lesions, as shown by the T_2_-weighted images and in revealing the entire affected tracts (Yeh et al., 2013a). Overall it is a way to identify tracts with decreased or increased connectivity and tracking differences. The conventional tractography approach relies on defining tracts before comparing the difference, thereby making it vulnerable to issues related to crossing and branching patterns, reliability and reproducibility. Diffusion connectometry overcomes these problems by finding the local difference in diffusion distribution first without initiating fiber tracking. This therefore, filters out regions without a substantial difference, leaving only pertinent affected regions for further analysis. This approach is more robust for a number of reasons: (a) it has a higher chance of detecting real differences, as the analysis relies on analyzing the difference in diffusion pattern, close to the signal level of the scan; (b) the entire process is fully automated, therefore related variability from manual selection of the ROI is eliminated; and (c) the obtained results can be statistically tested using the length of the affected tracks as a threshold, as described later. Further details regarding this methodology is provided in Yeh et al. (2013a). Diffusion connectometry approach can be tailored to test both individual and group data against a control population. Results from this approach can then be examined further using a targeted tractography approach.

### IMAGING DATA

#### Image acquisition and reconstruction

Diffusion spectrum imaging data was acquired on a 3T Tim Trio System (Siemens, Erlangen, Germany) using a 32-channel coil. A head stabilizer was utilized to prevent head motion. A 43-min, 257-direction DSI scan with a twice-refocused spin-echo planar imaging sequence and multiple *b* values (repetition time = 9916 ms, echo time = 157 ms, voxel size = 2.4 mm × 2.4 mm × 2.4 mm, field of view = 231 mm × 231 mm, maximum *b*-value = 7000 s/mm^2^) was performed. For anatomical comparisons, we also included a high-resolution anatomical image using a 9-min T_1_-weighted axial magnetization-prepared rapid-acquisition gradient-echo sequence (repetition time = 2110 ms, echo time = 2.63 ms, flip angle = 8°, number of slices = 176, field of view = 256 mm × 256 mm, voxel size = 0.5 mm × 0.5 mm × 1.0 mm). In this study, we discarded any dataset that had head motion greater than one voxel deviation across the long scanning time (by comparing the first b0 and last b0 image). For this preliminary study, we only selected patients who tolerated the scanning time well.

Diffusion spectrum imaging data is reconstructed with a generalized q-sampling imaging (Yeh et al., 2010) approach. The orientation distribution functions were reconstructed to 642 discrete sampling directions for each pixel and with a diffusion distance scaling parameter of 1.2. The resulting reconstructed orientation distribution function can cover 1.2 standard deviations of the free water diffusion distance in the underlying voxel (measured in micrometers). Similar acquisition parameters are being used to acquire control data to create a more accurate matched template file. Acquisition parameters for the template file, used for the connectometry analysis for the preliminary results, were detailed in previous publications, including in its previous use in the stroke study (Yeh and Tseng, 2011; Yeh et al., 2013a). The normal template, used for the current analysis (DSI studio: http://dsi-studio.labsolver.org) was derived from the DSI data of 90 neurologically normal subjects (Yeh and Tseng, 2011).

#### Step 1: diffusion connectometry analysis (Yeh et al., 2013a)

***Reconstructing spin distribution functions in a common space***. The diffusion data of each subject is reconstructed in a common stereotaxic space using q-space diffeomorphic reconstruction (QSDR; Yeh and Tseng, 2011), a method that satisfies the conservation of diffusible spins and can be applied to diffusion datasets. QSDR uses diffusion signals to calculate the spin distribution function (SDF). The DSI data of a studied subject, for example an ALS patient is also reconstructed by QSDR to obtain the SDFs in the MNI space. Further details regarding the reconstruction of SDF in a common space can be found in our original methodology paper (Yeh et al., 2013a).

***Constructing the affected tracks***. To demonstrate the segment of the tracks that are affected by ALS, we performed fiber tracking on the local tract orientations with a percentile rank lower than 5 (**Figures 2**–5), where local tract orientations with diffusion quantities (SDF values) lower than fifth percentile rank are plotted with directional colors (red: left–right, green: anterior–posterior, blue: superior–inferior), and tracks propagate along those local tract orientations. The tracking is conducted using a deterministic fiber tracking algorithm in the DSI Studio. The tracking begins from each local fiber orientation as seeds and propagates until no orientation is found in the propagation direction. A maximum turning angle of 60° is used with a step size of 1 mm (i.e., half of the spatial resolution in the template space). The obtained trajectories, termed the affected tracks can be used to reveal the pathways with decreased connectivity. It is noteworthy that only local tract orientations with substantial loss in diffusion quantities are tracked. The affected tracks reveal only affected segments of the entire track pathways.

***Length of affected tracks***. We used the length of affected tracks (unit in voxel distance) to differentiate meaningful findings from random variations. The rationale behind it is that normal population may have false-positive affected tracks due to noise in diffusion weighted images, but its effect tends to appear randomly in space, resulting in fragmentation of the affected tracks. As a result, the distribution of the length of the affected tracks (termed length distribution) will be exponential since the concatenation of randomly distributed local tract orientations can be viewed as a Poisson process (Yeh et al., 2013a). In contrast, the true connectivity difference in patient group will continue along fiber pathways and form longer affected tracks. Using length as an index, the difference between random effect and true connectivity difference can be differentiated and statistically tested. We can select the affected tracks with length greater than a threshold (termed length threshold) as positive findings because these trajectories are most likely related to the disease. A higher length threshold may filter out most of the false discoveries but may not be sensitive enough to detect subtle difference. The determination of the length threshold is a trade-off between sensitivity and specificity. To determine a reasonable value, we can quantify the Type 1 error of a length threshold using false discovery rate (FDR).

**FIGURE 1:**
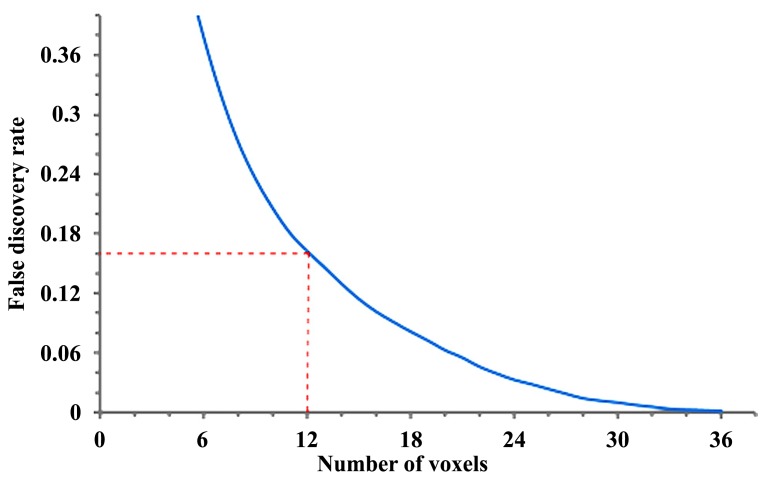
**Length threshold as defined by number of voxels in relation to the “false discovery rate” (FDR; each voxel = 2 mm).** A length threshold of 24 mm corresponding to 12 voxels was chosen, therefore leading to an FDR of 0.16.

***Adaptation of the original methodology and calculation of false discovery rate***. The calculation of FDR requires the length distribution calculated from a group of subjects. In a previous study (Yeh et al., 2013a) and here with the preliminary results, we obtained the affected tracks of each patient by comparing his/her data with those of 90 normal subjects (Yeh and Tseng, 2011). The length of affected tracks in all patients is accounted to obtain the empirical length distributions for the patient group. Similarly we can treat normal subjects as patients and apply the same analysis to each of them by comparing his/her data with those of the other 89 normal subjects to obtain the empirical length distribution for the normal population. The difference in the affected tracts between patient and normal subject groups can be used to calculate the FDR of our findings in the ALS patients (Yeh et al., 2013a). In step 1 (**Figure 1**), using a fifth percentile ranking, we regarded any affected track with length >24 mm as related to ALS, giving us an FDR of 0.16. This means that 16% of the affected tracks may be false discoveries. Length threshold and the FDR provide a quantitative measurement of sensitivity/specificity of a finding. In order to track longitudinal data, we used the same FDR and length threshold to examine the subsequent scan, with the hypothesis that any demonstrated progression in the affected tracts is an indirect marker of disease progression.

#### Step 2: HDFT and targeted fiber tractography

***Fiber tracking analysis***. For the HDFT data, fiber tracking was performed with DSI Studio. Tracts are generated with the use of an orientation distribution function-streamlined version of the fiber assignment by continuous tracking (FACT) algorithm (Basser et al., 2000; Yeh et al., 2010). Fiber tracking is initiated by randomly seeding the ROI and initiating tracking in the direction of the most prominent fiber. Fiber progression continues with a step size of 1.2 mm, minimum fiber length of 20 mm, and turning angle threshold of 65°. For smoothing of the tracts, the next directional estimate of each voxel is weighted by 20% of the previous moving direction and 80% by the incoming direction of the fiber. The tracking terminates when the relative QA for the incoming direction drops below a preset threshold or exceeds a turning angle of 65°. The QA termination threshold is adjusted on a per-subject basis, so that the threshold allows consistent white matter coverage in all subjects. This is critical for single-subject-based tractography studies in which individual differences in coil sensitivity and diffusion signal homogeneity can vary across subjects. After tracking, all streamlines were saved and tract segmentation performed in DSI Studio. The QA threshold is kept constant across the longitudinal scans for the same subject. Mean QA and volumes (voxel wise) are then obtained for affected fiber tracts of interest, identified from connectometry analysis for both hemispheres. This is followed by calculation of percentage changes in QA and volume across longitudinal scans in order to obtain quantitative data with respect to progression in order to enable potential correlation with markers of clinical progression. Obtained data was also evaluated qualitatively in terms of obvious “thinning” of the tracts in a cross-section or discontinuity in a tract segment.

## RESULTS

### STEP 1: DIFFUSION MRI CONNECTOMETRY ANALYSIS

In order to track progression with longitudinal data in patients 1 and 2, and for current non-longitudinal data in 3, 4, and 5 we chose a length threshold of 24 mm (corresponding to 12 voxels), thus giving us an FDR of 0.16 (**Figure 1**) for the observed affected tracts. This threshold was kept constant across longitudinal scans to detect progressive changes with disease evolution.

#### Patient 1

**FIGURE 2:**
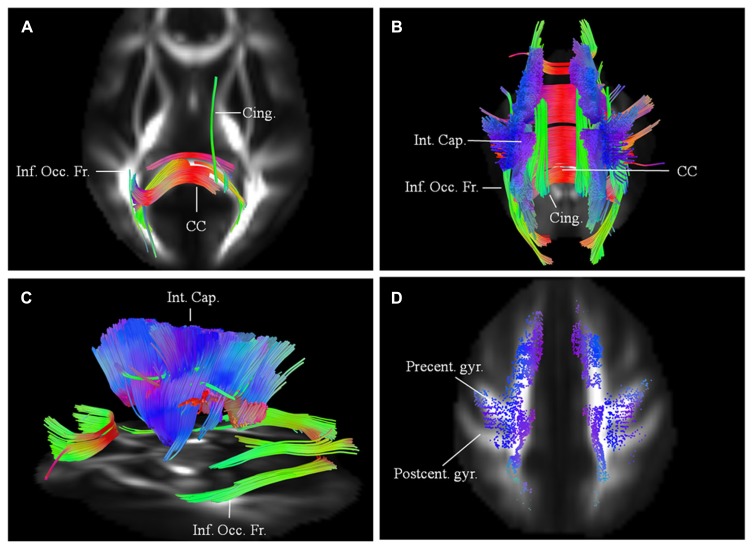
** (A)** Baseline diffusion connectometry analysis for patient 1, demonstrated the affected areas as being inferior occipito-frontal fasciculus (Inf. Occ. Fr.), cingulum (Cing.), and posterior part of the corpus callosum (CC). **(B**, posterior view) and **(C**, lateral view) a year later, extensive changes are now evident bilaterally in the corpus callosum; cinguli and posterior limb of the internal capsule (Int. Cap.) and in parts of inferior occipito-frontal fasciculi. **(D)** Cortical end-points of the affected fiber tracts at the time of the second scan demonstrate involvement of fibers originating from precentral gyrus (Precent. gyr.) and frontal areas with relative sparing of postcentral gyrus (Postcent. gyr.).

Results are presented for this 48-year-old patient with a diagnosis of probable laboratory supported ALS (limb onset; predominant LMN involvement) with respect to two scans acquired approximately 1 year apart. His symptoms started about 30 months before the baseline scan with widespread fasciculations and left hand weakness. He has no family history of neurological disease. Diffusion connectometry analysis revealed the changes predominantly at the baseline in posterior part of the CC (**Figure 2A**). A year later (**Figures 2B–C**), these changes had progressed to include the fibers in the posterior limb of the internal capsule (IC) predominantly, CST and frontal fibers coursing toward the brainstem; cingulum, and other areas. These changes were consistent with multisystem neurodegeneration and frontal progression with disease evolution. The patient’s ALSFRS-R score decreased from 45 to 32 during this interval. Cortical end points of the involved fiber tracts confirmed relative sparing of the postcentral gyrus and associated sensory fibers (**Figure 2D**), consistent with known ALS pathology.

#### Patient 2

**FIGURE 3:**
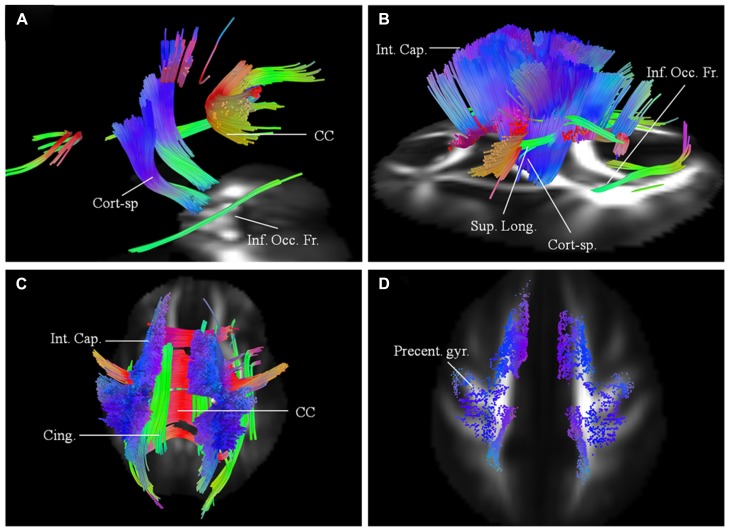
**(A)** Baseline diffusion connectometry analysis for patient 2, demonstrated the affected areas as being parts of the corpus callosum (CC), corticospinal (Cort-sp.) tracts, and inferior occipito-frontal (Inf. Occ. Fr.) fasciculi bilaterally. **(B**, left lateral view) and **(C**, posterior view) 6 months later shows extensive bilateral changes now involving corticospinal tracts, frontal fibers coursing toward the brainstem in the posterior limb of the internal capsule (Int. Cap.); cinguli (Cing.); superior longitudinal (Sup. Long.); and inferior occipito-frontal fasciculi. **(D)** Cortical end points for the second scan display predominant involvement of fibers originating from the precentral gyrus (Precent. gyr.) and frontal areas.

Imaging results for this 62-year-old male patient with probable ALS (bulbar onset with predominant bulbar involvement) with respect to two scans carried out approximately 6 months apart are presented. His symptoms started with dysarthria. The disease duration at the time of the baseline scan was 12 months. He has no family history of neurological disorders. At baseline (**Figure 3A**), the affected white matter tracts were predominantly the anterior and posterior parts of the CC and parts of the CST bilaterally. CST was involved more caudally than in patient 1. At the second scan, 6 months later (**Figures 3B–C**), the changes in white matter were more extensive involving the CC; corticostriatal fibers; fibers in the posterior limb of IC, including CST and the frontal fibers coursing toward the brainstem; cingulum and parts of the superior longitudinal (SLF) and inferior occipito-frontal fasciculi (IOF) bilaterally. These findings were more pronounced on the left side. Over the scan interval, his ALSFRS-R score dropped from 44 to 43. Cortical terminations of the affected fibers, as in patient 1 showed frontal progression with disease evolution (**Figure 3D**).

#### Patient 3

**FIGURE 4:**
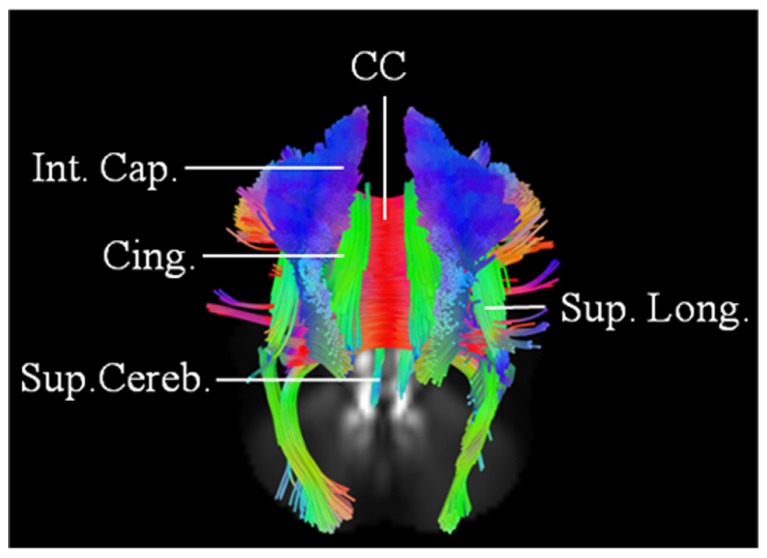
**Baseline scan (posterior view) in patient 3, demonstrating evidence of extensive motoric and extramotoric changes in the white matter tracts [corpus callosum (CC); internal capsule (IC); Superior longitudinal (Sup. Long.) fasciculus; Cingulum (Cing.)] with changes being more pronounced on the right; also noted was involvement of the left superior cerebellar peduncle (Sup. Cereb.)**.

This 62-year-old male patient with probable ALS (limb onset), has undergone a single scan, so far 24 months into his disease onset, characterized by left hand weakness and foot drop. He has no family history of neurological disorders. The baseline scan demonstrated extensive involvement of the CC; CST; SLF; IOF; and cingulum bilaterally. The changes were more pronounced on the right. There was also evidence of involvement of left superior cerebellar peduncle (**Figure 4**). ALSFRS-R score at baseline scan was 43.

#### Patient 4

**FIGURE 5:**
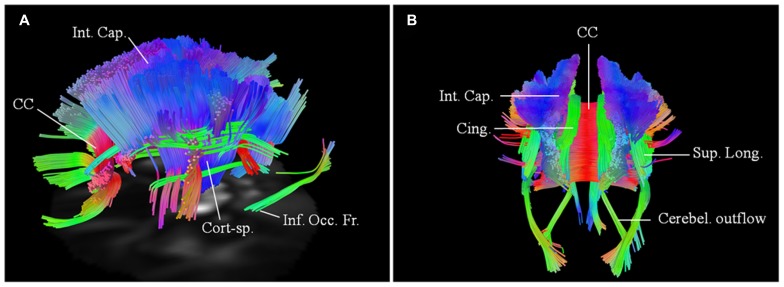
**Baseline scans in patients 4 (A, left lateral view) and 5 (B, posterior view) demonstrating bilateral changes in the motoric and extramotoric pathways, being more pronounced on the right**. Bilateral involvement of the cerebellar outflow tracts (Cerebel. outflow) was noted in patient 5. Bilaterally involved tracts in both included corpus callosum (CC); corticospinal tract (Cort-sp.); frontal fibers in the posterior limb of the internal capsule (Int. Cap.) coursing toward the brainstem; cinguli (Cing.) and parts of superior longitudinal (Sup. Long.) and inferior occipito-frontal (Inf. Occ. Fr.) fasciculi.

A 62-year-old with probable ALS-laboratory supported (limb onset with predominant UMN involvement) was 38 months into her disease onset at her baseline scan. ALSFRS-R score was 40 at the time of the baseline scan. Her initial symptoms were clumsiness of the left foot and leg. There was no family history of ALS, but mother had Alzheimer’s disease. Diffusion connectometry analysis (**Figure 5A**) demonstrated changes in motoric and extramotoric tracts. As observed with other patients, changes were seen bilaterally (right > left) in CST, CC, cingulum, parts of SLF, and IOF (**Figure 5A**).

#### Patient 5

A 74-year-old female has possible ALS (bulbar onset with predominant UMN signs and bulbar involvement). Her symptoms started about 27 months before the baseline scan with dysarthria. She has no family history of neurological diseases. Baseline scan demonstrated similar changes, as in patient 4 with more pronounced involvement of the tracts on the right side. There was however a greater rostro-caudal involvement of the CST, with associated involvement of the cerebellar outflow tracts bilaterally (**Figure 5B**).

### STEP 2: TARGETED FIBER TRACKING USING HDFT OF AFFECTED TRACTS FROM DIFFUSION CONNECTOMETRY

**FIGURE 6:**
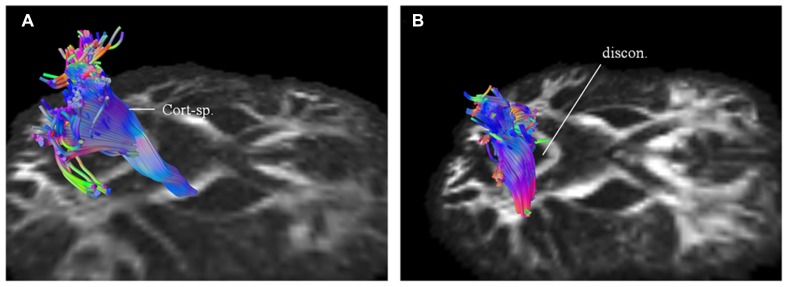
**Targeted fiber tracking in patient 1 demonstrated evidence of progressive compromise of the right corticospinal tract (Cort-sp.) between the baseline scan (A) and the subsequent scan (B), a year later, on which discontinuity (discon.) in the caudal segment was noted**.

Using patients 1 and 2 with longitudinal scans, we calculated the changes in QA and volumes for selected major white matter tracts, affected through majority of their course and as identified from the connectometry analysis (**Table 1**). As an example for patient 1, we selected CST for closer evaluation using changes in QA and volume over 1 year scan interval. With similar tracking parameters and QA threshold for the two scans, there was progressive compromise of the CST bilaterally (**Figure 6**; right CST). This was seen qualitatively by presence of discontinuity in its caudal part and quantitatively by percentage decreases in QA for the left and right at 25% and 28% respectively and associated percentage volume reductions at 5% and 41% respectively.

**Table 1:** Changes in quantitative markers in two patients with longitudinal scans for major affected white matter tracts, as identified by the connectometry analysis.

Patient number (scan interval)	Percentage changes^*^ in quantitative markers	Affected major white matter tracts				
		L CST	R CST	CC	L cingulum	R cingulum
1 (12 months)	QA	-25%	-28%	-20%	-23%	+16%
	Volume	-5%	-41%	-86%	-73%	-71%
2 (6 months)	QA	-15%	-17%	+5%	-4%	+8%
	Volume	-61%	-72%	-67%	-78%	-39%

* :the percentage changes denote a decrease (-) in all cases except those denoted by the sign (+), where an increase was noted; CST, corticospinal tract; CC, corpus callosum; L, left; R, right; QA, quantitative anisotropy.

## DISCUSSION

In her paper published, Smith (1960) aimed to study the degeneration of myelinated fiber tracts in ALS. She demonstrated wider extramotor involvement, including frontal cortex in addition to the CST and CC. Further involvement of fibers projecting to the thalami and basal ganglia were also noted. In this remarkable early study (Smith, 1960) pathological involvement of both motoric and extramotoric white matter pathways in ALS was highlighted. We can now demonstrate these patterns *in vivo* using MR-based tractography techniques. White matter involvement is a subject of investigation in both longitudinal and non-longitudinal DTI studies in ALS toward assessing its use as a diagnostic tool, including in distinguishing between different phenotypes based on UMN versus LMN involvement and in monitoring progression.

Longitudinal studies are useful toward demonstrating the time course and progression of pathology in ALS. A longitudinal study involving 24 subjects with ALS demonstrated a decrease in the FA in the CST and frontal areas on a second scan, obtained 6 months later (Keil et al., 2012). The progressive FA decrease along the CST correlated with disease duration and the decrease in ALSFRS-R (Keil et al., 2012). Similarly another study with 6 months follow-up showed DTI abnormalities extending into bilateral frontal lobes (Senda et al., 2011). van der Graaff et al. (2011) examined the three phenotypes, i.e., ALS, PLS and progressive muscular atrophy (PMA) within the motor neuron disease (MND) spectrum. This study assessed the upper- and extra-motoneuron white matter involvement shortly after diagnosis by comparing DTI data of the different cohorts to those of healthy controls and by directly comparing disease phenotypes. Follow-up data was also acquired 6 months later to evaluate FA changes over time. Diffusion tensor tractography of the CST and whole-brain VBA was utilized to ascertain the white matter involvement in MND. VBA demonstrated white matter involvement to varying extents in different phenotypes although in quite similar anatomical locations. In general, FA reductions were found to be most extensive in PLS with the observation of both upper motor and extra-motoneuron involvement in all phenotypes of MND shortly after diagnosis. VBA was more sensitive in detecting longitudinal changes than tractography of the CST. VBA was also thought to be particularly valuable in the evaluation of motor and extra-motor white matter involvement in the early symptomatic MND, and in monitoring the spread of pathology over time (van der Graaff et al., 2011). We have focused our current efforts toward monitoring and establishing the nature of disease progression in the white matter via a longitudinal study. In the future, we will aim to assess its use as a diagnostic tool with potential value in distinguishing different MND phenotypes.

### CURRENT FINDINGS AND RELEVANCE OF PRELIMINARY DATA

Based on preliminary results presented here, including in two patients with longitudinal scans, we demonstrate a number of features that are consistent with pathological studies (Smith, 1960), including presence of disease in the motor pathways (Ellis et al., 1999; Sach et al., 2004; Iwata et al., 2008; Sage et al., 2009), IC and CC (Filippini et al., 2010). Beyond the motor system, we demonstrate changes in the limbic pathways (Sarro et al., 2011; Keil et al., 2012); spread of the disease frontally (Ellis et al., 2001; Keil et al., 2012) and potential involvement of the cerebellum (Kassubek et al., 2005; Keil et al., 2012). Cerebellar involvement has been implicated in previous functional studies (Schoenfeld et al., 2005; Konrad et al., 2006). Involvement of the CC, which connects orbitofrontal and frontal cortices, is not surprising in ALS and has been deemed to be a fairly consistent feature (Filippini et al., 2010). Similarly involvement of frontal white matter and cingulum may contribute to the cognitive symptoms in ALS patients (Abrahams et al., 2005; Raaphorst et al., 2011; Sarro et al., 2011; Keil et al., 2012). Cerebellar outflow tract involvement as seen in patient 5 has been postulated (Schoenfeld et al., 2005; Konrad et al., 2006; Keil et al., 2012) to be related to functional compensation; however the exact relevance of its altered connectivity and structural impairment is unclear. Although cerebellar signs are lacking in most ALS patients, previous pathological (Swash et al., 1988) and functional MRI studies have suggested cerebellar involvement. The implication of cerebellar involvement is typically attributed to functional compensation in ALS rather than development of new synapses (Schoenfeld et al., 2005).

Functional correlation in this initial dataset is difficult owing to a small current size, however in both patients 1 and 2 with longitudinal scans and evidence of imaging progression of white matter disease, there was decline in an already compromised ALSFRS-R score. This was more pronounced in patient 1 with a longer interval between the scans. It is noteworthy that patient 2 demonstrated less extensive changes on the baseline scan in comparison with other cases. This is likely related to the first scan being undertaken earlier in the disease course. Future analysis of the larger dataset will shed light on a potential correlation between observed imaging changes and rate of decrease in ALSFRS-R scores, including how baseline values for functional scores relate to the initial scan. Patient 2, for example, had a similar functional score to patient 5 with widespread white matter involvement, as seen on the connectometry analysis, but had less extensive imaging changes. One potential explanation for this is the inherent difficulty of correlating observed imaging changes in the UMN with ALSFRS-R, which is a composite score relying on multiple neuronal pathways and LMN. Another explanation relates to the qualitative nature of information derived from connectometry analysis. A further explanation pertains to the heterogeneity in potential functional compensation between ALS patients with similar possible disease burden. These partly explain a further need to validate the findings from connectometry analysis with targeted tractography approach, so that quantitative markers, for example changes in QA may be examined in relation to functional scores. Another finding needing future investigation is asymmetry of radiological changes, as demonstrated here and their potential correlation with the clinical findings. This is observed in both patients 3 and 4, where more extensive imaging changes were found on the right side on their baseline scan with the disease onset occurring on the left side.

### LIMITATIONS, FUTURE EXPERIMENTAL PLAN, AND DIRECTIONS

Another methodology, apparent fiber density (AFD; Raffelt et al., 2012) compares the difference in fiber orientation distribution and uses a clustering approach to group voxels with significant differences. Since the clustering is voxel-based, it does not address differences from different fibers within the same voxel. The clustering algorithm may cluster unrelated differences. Connectometry avoids this problem, as it is fiber-based, and the differences in multiple fibers are considered separately using a fiber skeleton. Further, AFD is based on the spherical deconvolution method, which is limited to HARDI, and has been shown to be subject to CSF contamination. Our connectometry method can make use of both the DSI and HARDI data, and the partial volume effect is corrected using the spin density information.

One limit in the current study is the small sample size. We have however shown that our previously presented analytical method (Yeh et al., 2013a) may be adapted to test longitudinal data in ALS. In this respect, the current study presents preliminary results. The 40-min DSI scan can now be replaced by multi-band DSI sequence (Sotiropoulos et al., 2013), which takes less than 10 min to acquire half sphere 101-directions DSI. This will dramatically improve the clinical applicability of this method, particularly for patients with neurodegenerative disorders. We are currently in the process of deploying this sequence for scanning new patients.

In addition to the previously mentioned details, we will examine the relationship between clinical and imaging progression on connectometry analysis. We will further corroborate the findings from connectometry analysis by performing targeted analysis of the affected tracts beyond CST for example, cingulum or thalamopostcentral fibers. We will therefore, aim to further identify tracts, that may be preferentially affected and calculate the changes in the associated quantitative markers. The changes in these will be correlated with the rate of disease progression, for example drop in ALSFRS-R scores with change in CST or decline in ALS cognitive behavioral screen with change in cingulum. In future work, we will need to establish a normal range of expected variability in QA and/or volumes for fiber tracts of interest between scan sessions for comparison with observed changes in affected fibers. Although with disease progression, a decrease in mean QA will be expected for a specific fiber tract of interest, the increase as observed for a few tracts (**Table 1**) can be either explained by the variability in QA across scan sessions or potential functional compensation of the remaining fibers. This remains to be elucidated further in the future. Similarly, variability issues with respect to the calculated volumes may accentuate the extent of the findings in terms of disease progression. This may explain the large noted percentage decrease in the volume of the callosal fibers in patient 1. We will also explore and establish an optimal length threshold and the associated FDR for ALS patients, particularly in relation to our matched control dataset.

One of the endeavors associated with DTI in ALS has been evaluation of its use as a diagnostic tool and as an aid enabling discrimination between different MND phenotypes. A recent meta-analysis (Foerster et al., 2013) concluded that DTI analysis in ALS only had modest discriminatory capability. Further, there were no significant differences in the diagnostic test accuracy estimates with respect to MRI field strength or brain location. Potential reasons for this poor result may be the clinico-pathological heterogeneity in ALS, and use of group studies. These issues become even more pertinent in the context of different MND phenotypes. Although there is increasing evidence that the different MND phenotypes lie within a spectrum (van der Graaff et al., 2011; Agosta et al., 2014) with overlapping white matter abnormalities, the anatomical location and the extent of these changes vary, making group comparisons difficult. This is the primary reason we have focused on ALS patients’ individual progression and have started by exploring the nature, extent and distribution of findings from the connectometry analysis. Having established these we will embark upon further studies applicable toward diagnosing and differentiating between different MND phenotypes. This could be achieved, for example, by using the varying anatomical (rostro-caudal) and quantitative involvement of CST as a guide.

In conclusion, we have presented preliminary results, using DSI in longitudinal evaluation of white matter pathology in ALS, as a first step toward developing an imaging biomarker to monitor disease progression. We ultimately intend to establish an imaging biomarker capable of evaluating efficacy of novel therapies in slowing or reversing the disease progression.

## AUTHOR CONTRIBUTIONS

Kumar Abhinav made substantial contributions to the conception of the idea, design, data analysis and interpretation of the work in addition to drafting the manuscript; approving the final version to be published and being accountable for the all aspects of the work. All remaining authors contributed to these aspects; however specific contributions, as indicated in brackets were: Fang-Cheng Yeh (review of results and study design); Ahmed El-Dokla (clinical data acquisition and interpretation); Lisa M. Ferrando (clinical and imaging data acquisition); Yue-Fang Chang (statistical input); David Lacomis (clinical data acquisition; overall interpretation and critical appraisal); Robert M. Friedlander (conception of the idea, overall interpretation, and critical appraisal); Juan C. Fernandez-Miranda (conception of the idea, study design, overall interpretation, and critical appraisal of manuscript).

## Conflict of Interest Statement

The authors declare that the research was conducted in the absence of any commercial or financial relationships that could be construed as a potential conflict of interest.
